# When I Am Asked: Lisel Mueller’s Beginning in Poetry

**DOI:** 10.14797/mdcvj.1335

**Published:** 2024-03-14

**Authors:** Justin C. Cordova, James B. Young

**Affiliations:** 1 Department of Anesthesiology, Walter Reed National Military Medical Center, Bethesda, Maryland, US; 2 Cleveland Clinic, Cleveland Clinic Lerner College of Medicine of Case Western Reserve University, Cleveland, Ohio, US

## Abstract

Why does anyone write poetry? Lisel Mueller (1924–2020) was a poet, author, and translator with a long and much-decorated career. She and her family fled Nazi Germany in the 1930s and emigrated to the United States, where she would establish herself as a writer. The poem “When I Am Asked” describes the beginning of her journey into poetry, undertaken during a period of grief after the death of her mother. Her writing would come to include nine collections of poetry and myriad accolades, including the 1981 National Book Award and the 1997 Pulitzer Prize for Poetry. Though her ouvre is filled with evocative works, this piece stands out as particularly relevant to physicians and other writers who find solace by expressing themselves through the art of poetry.

## When I Am Asked

When I am asked
how I began writing poems,
I talk about the indifference of nature.

It was soon after my mother died,
a brilliant June day,
everything blooming.

I sat on a gray stone bench
in a lovingly planted garden,
but the day lilies were as deaf
as the ears of drunken sleepers
and the roses curved inward.

Nothing was black or broken
and not a leaf fell
and the sun blared endless commercials
for summer holidays.

I sat on a gray stone bench
ringed with the ingenue faces
of pink and white impatiens
and placed my grief
in the mouth of language,
the only thing that would grieve with me.

“When I Am Asked” from *Alive Together: New and Selected Poems*. Copyright © 1996 by Lisel Mueller. Reprinted by permission of Louisiana State University Press.

## Commentary

When Ilse Neumann passed away in June 1953, her daughter was stricken with a grief for which she could not find an outlet. She eventually turned to poetry, joining the ranks of other notable women poets. Thus began the historic writing career of Lisel Mueller. Her work would span almost four decades—producing nine volumes of poetry and prose, several works in translation, and ultimately culminating in the Pulitzer Prize for Poetry in 1997. “When I Am Asked” is a seminal piece in her ouvre, as it demonstrates the motivations behind her work while also leading its readers to question their own motivations for expressing themselves through verse. This is particularly relevant to healthcare providers and their motivation to pen poetry. Though Mueller passed away in February of 2020, her words will outlive her, continuing to impact the world of literature today and into the future.

**Figure:**
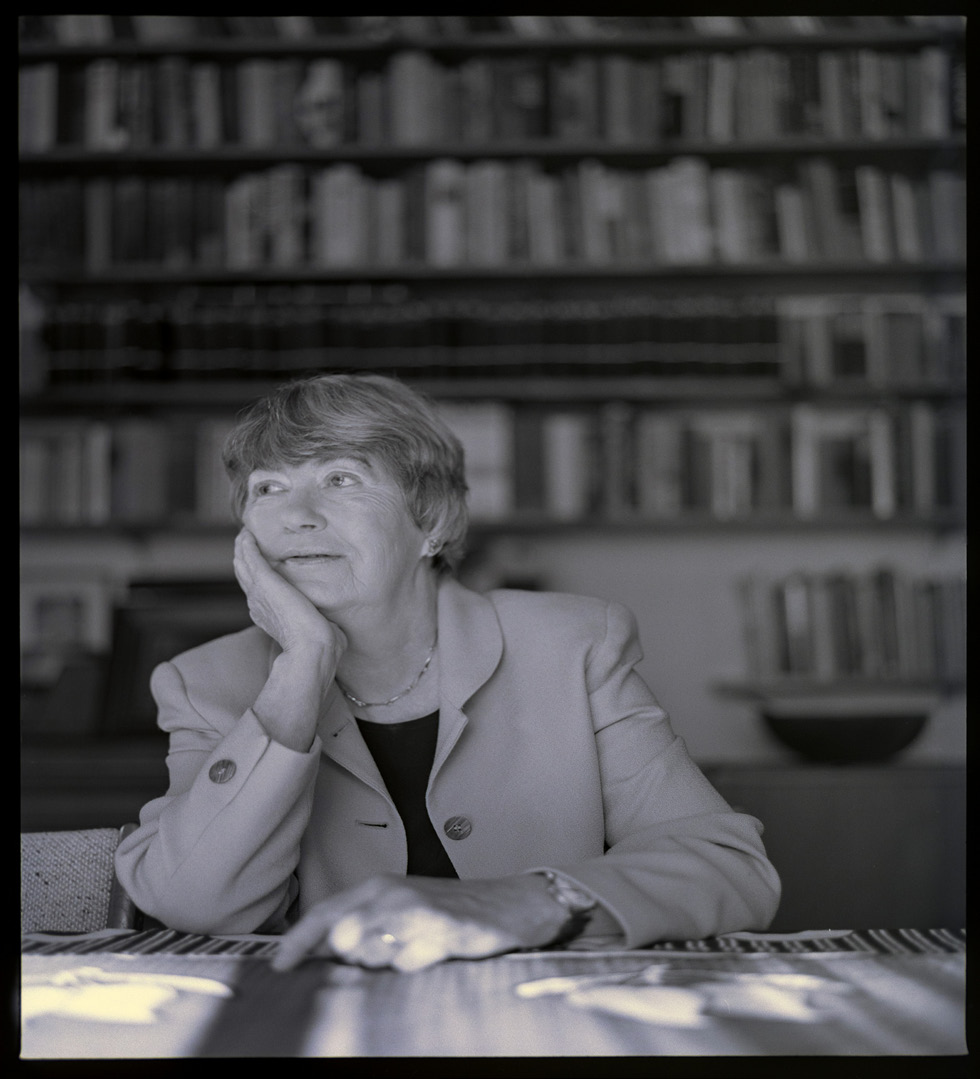
Poet Lisel Mueller; photo by Tom Maday.

Lisel Mueller was born on February 28, 1924, as Elisabeth Annelore Neumann in Hamburg, Germany. Her father, Fritz Neumann, was a teacher with anti-fascist political sentiments who was forced to flee the country in 1933, shortly after Adolf Hitler and the Nazi regime came to power.^1^ Her mother, Ilse, was also a teacher and would join her husband in 1939 along with Lisel and her sister, shortly before Germany invaded Poland and the Second World War began.^2^ The family settled in Evansville, Indiana, home of Evansville College, where Fritz gained a professorship and Lisel would later attend as an undergraduate. While there, she was deeply influenced by the poetry of John Keats and Carl Sandburg,^3^ though her own writing journey would not begin until much later. Before graduating with a degree in sociology, Lisel met and married her husband, Paul Mueller. After graduation, she underwent postgraduate studies in comparative literature at the University of Indiana before she and Paul relocated near Chicago.^4^

Once in Illinois, Lisel worked as a social worker and librarian while also reviewing poetry for the *Chicago Daily News* and *Poetry* magazine. She would go on to teach at Elmhurst College and the University of Chicago and was on faculty in the Master of Fine Arts program that began at Goddard College and later moved to Warren Wilson College.^5^ Though her first volume of poetry was not published until she was 41, Mueller’s life and work would slowly accrue the awards and recognition that it garners today. To name but a few of her accolades, she was awarded the Lamont Poetry Selection of the American Academy of Poets for *The Private Life* in 1975, won the 1981 National Book Award for her poetry collection *The Need to Hold Still*, and was awarded the Pulitzer Prize for Poetry in 1997 for *Alive Together: New and Selected Poems*. In this penultimate achievement, Lisel joined the likes of Robert Frost, Sylvia Plath, and William Carlos Williams, becoming the first German-born author to do so. In 2002, she would also receive the Poetry Foundation’s Ruth Lilly Poetry Prize for lifetime achievement.^6^

As Mueller describes in “When I Am Asked,” her historic career as a poet began with the death of her mother in 1953. She told the *Chicago Tribune* that, “The great grief made me want to express myself in a poem and having done that I needed to continue to do so.” The poem, which typifies her juxtaposition of the natural with the cataclysmic and of the temporary with the permanent, was originally published as part of her sixth volume of poetry, *Waving from Shore* (1989). It was later reprinted as part of her Pulitzer Prize-winning final volume *Alive Together: New and Selected Poems* (1996), alongside many of her other popular pieces like “Curriculum Vitae,” “In Passing,” and “Palindrome.”

Mueller’s work continues to be accessible, as she used simple and concise language to respond to universal themes encountered in her daily life. As an immigrant, wife, and mother of two who lived in a rural setting, she wrote on parenting and nature, birth and aging, life and loss. She used poetry to respond to the world around her, recording her reactions to literature (“Another Version,” “Reader”), the arts (“Monet Refuses the Operation,” “Silence and Dancing”), history (“Beginning with 1914”), and the playfulness of language (“Things,” “The Possessive Case”). She wrote of life events that her readers could relate to and in a way that solidified her standing as a poet with a lasting impact upon posterity. She helped to codify poetry as a means for writers to respond to tragedy, to question their place in the present and future, and to consider their own motivations to put pen to paper and use verse as their voice.

Mueller died in 2020 at the age of 96, but her impact on the world of poetry continues through the students she taught, through her daughters—one of whom has published two volumes of poetry herself^7^—and through new readers who found her work long after she retired. Readers of her poetry and writers inspired by her works are ever grateful for her decision to “place her grief in the mouth of language.” Mueller is a role model for those in healthcare professions seeking literary forbearance to counter challenges of a demanding profession.
